# Presence and function of small RNAs during plant reproduction

**DOI:** 10.1080/15476286.2026.2679879

**Published:** 2026-05-26

**Authors:** Ann S. Yang, Alexander M. Tang, Mary Gehring

**Affiliations:** a Whitehead Institute for Biomedical Research, Cambridge, MA, USA; b Department of Biology, Massachusetts Institute of Technology, Cambridge, MA, USA; c Department of Biological Engineering, Massachusetts Institute of Technology, Cambridge, MA, USA; d Howard Hughes Medical Institute, Whitehead Institute for Biomedical Research, Cambridge, MA, USA

**Keywords:** seed development, small RNAs, RNA Pol IV, Genome dosage, RNA-directed DNA methylation

## Abstract

Plants possess an expansive suite of epigenetic control mechanisms to keep their large, repetitive, and unruly genomes in check. Small non-coding RNAs constitute one such mechanism by directing transcriptional and post-transcriptional gene silencing. Small RNAs come in several forms that are produced by distinct pathways, including microRNAs and small interfering RNAs, such as tasiRNAs, phasiRNAs, and RNA Polymerase IV (Pol IV)-dependent small RNAs. Compared to other classes of small RNAs, Pol IV-dependent small RNAs and phasiRNAs appear to have the greatest phenotypic impact during the reproductive phase of the life cycle, from meiosis through seed development. Here, we focus on the function of 21–24 nucleotide small RNAs during reproduction, with a primary focus on Pol IV-dependent small RNAs. We critically assess the evidence surrounding their mode of action, specifically whether they act in *cis* or in *trans*, cell autonomously or non-autonomously, and within or between generations.

## Introduction

**Figure 1.**
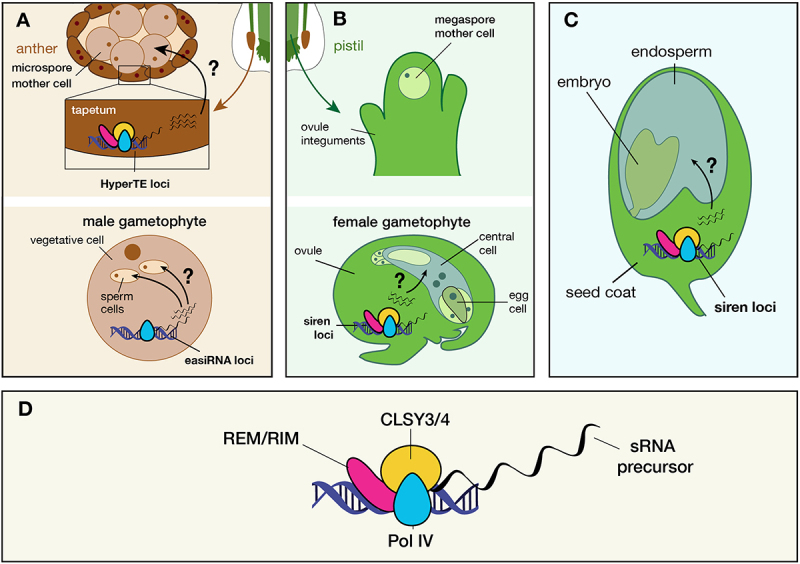
Sexual reproduction and the potential movement of Pol IV-dependent sRNAs. (A) Prior to male gametogenesis, CLSY3 and REM/RIM transcription factors recruit Pol IV to HyperTE loci in tapetal cells to transcribe sRNAs. These sRNAs are proposed to move to and function in the microspore mother cells. After mitosis, the male gametophyte (pollen) accumulates Pol IV-derived 21–22-nt sRNAs over easiRNA loci. It is currently unclear if CLSYs or other transcription factors recruit Pol IV in this context. (B) During female gametogenesis, the megaspore mother cell undergoes a series of meiotic and mitotic divisions to form the mature seven-celled female gametophyte. In ovules, Pol IV is recruited to siren loci in a CLSY3/4 and REM/RIM dependent manner and generates sRNAs that match TEs and imperfectly match protein-coding genes. It has been proposed that sRNAs from maternal tissues might translocate into the female gametophyte, which contains the gametes: egg and central cell. (C) Post-fertilization, CLSY3/4 and REM/RIM factors likely function in the seed coat to generate sRNAs. Some models propose that these sRNAs move into the endosperm, although direct evidence of movement is lacking. Not shown are the abundant Pol IV-dependent 24-nt sRNAs produced in the embryo and endosperm from typical loci. Post-germination, Pol IV remains active in vegetative tissues and is primarily dependent on recruitment by CLSY1/2 and SHH1 to transposable elements (not shown). Question marks in A-C indicate proposed movement of sRNAs or their precursors. (D) CLSY3/4 (yellow) recruits Pol IV (blue) to genomic loci in a REM/RIM (pink) dependent manner. At these loci, the Pol IV complex transcribes sRNA precursors (black).

A diverse array of small non-coding RNAs (sRNAs) are abundant in plants and are defined by their mode of biogenesis, size, sequence content, and molecular function [1]. Twenty-four nucleotide (nt) sRNAs are the most abundant size class in angiosperms, with the majority matching transposable elements (TEs), TE-related sequences, and tandem repeats. 21–23 nt sRNAs are also typically produced from the same regions as 24-nt sRNAs but in lower abundance. In this review, we consider the function and consequences of sRNAs and the processes that produce them during reproduction in flowering plants. We define reproduction as beginning at the specification of meiotic cells through gametophyte development, continuing through fertilization and ending at the completion of embryo and endosperm development (Figure 1). The most is known about 24-nt sRNAs that are dependent on RNA Polymerase IV (Pol IV), but we also briefly review RNA Polymerase II-dependent phasiRNAs, which are abundant in reproductive tissues of monocots and most eudicots.

Twenty-fournt sRNAs are produced when the enzyme DCL3 (DICER-LIKE3) cleaves a ~ 30–40-nt double stranded RNA (dsRNA) precursor that is transcribed and copied by Pol IV and the closely associated RNA-DEPNDENT RNA POLYMERASE2 (RDR2) [2]. The sRNAs produced from Pol IV-RDR2 transcripts can direct DNA methylation. During the process of RNA-directed DNA methylation (RdDM), 24-nt dsRNAs are loaded into an ARGONAUTE (AGO) protein (typically AGO4 or AGO6), and the passenger strand is cleaved, leaving the guide strand intact. RNA Polymerase V (Pol V) is responsible for generating long non-coding RNAs (lncRNAs), which act as scaffolds that bind to the guide strand in AGO, thereby recruiting the *de novo* DNA methyltransferase DRM2 and resulting in deposition of DNA methylation in all sequence contexts. Pol IV-RDR2 precursor transcripts can also be processed by DCL2 or DCL4 to produce 21–22-nt small RNAs that can direct DNA methylation or function in the cleavage of target RNA [3,4].

Methylated CG and CHG sites are maintained after DNA replication by maintenance methyltransferases, whereas methylated CHH sites are maintained by continual RdDM or by CHROMOMETHYLASE2 (CMT2). CMT2 maintains mCHH in long transposons within heterochromatin, via interaction with dimethylated lysine 9 of histone 3 (H3K9me2), in a process that also requires the chromatin remodeller DDM1, whereas DRM2 maintains mCHH at the ends of transposons and in more open regions of the genome. In complex, TE-rich genomes like that of maize and rice, RdDM activity is enriched at short mCHH islands that exist at the boundaries between active genes and TEs; this activity promotes TE silencing [5–7]. In contrast to Arabidopsis, maize DDM1 occupancy is also enriched at RdDM sites and DDM1 copurifies with AGO4, suggesting cooperation between the chromatin remodelling activities of DDM1 and RdDM [8].

Pol IV and Pol V are evolutionarily descended from and share many subunits with RNA Polymerase II (Pol II) but have distinct properties and functions [9]. Recently, cryogenic electron microscopy (cryo-EM) has determined the structure of the Pol IV-RDR2 holoenzyme and isolated components, shedding light on how the two RNA polymerases function together to create short dsRNA molecules [10–12]. Pol IV backtracking facilitates passing of its RNA product to RDR2 through an inter-polymerase channel, where it is used as a template for synthesis of the second strand. Cryo-EM has also been applied to cauliflower Pol V to elucidate the mechanism by which the Pol V catalytic subunit NRPE1 captures the non-template DNA strand to enhance backtracking and increase the fidelity of Pol V [13]. Pol IV is more error prone *in vitro* than Pol V, which could facilitate the production of sRNAs that can act in *trans* at multiple similar but not identical targets [14], which, as discussed below, might be particularly relevant in reproductive tissues. Another study used cryo-EM to determine the structure of Arabidopsis Pol V bound to the transcription factor KTF1, revealing the mechanism by which AGO proteins are recruited to Pol V to assemble the DNA methylation protein complex [15].

In addition to the canonical RdDM pathway, there are non-canonical RdDM pathways that result in deposition of DNA methylation without the involvement of DCLs or, in some cases, Pol IV [16]. For example, the dsRNAs made by Pol IV-RDR2 can be processed to maturity by 3’-5’ exonucleases, RRP6L1/ATRIMMER1 and ATRIMMER2 [17]. The non-canonical RDR6-RdDM pathway is important for initiating DNA methylation at previously naïve loci by incorporating transcripts made by Pol II or Pol II-RDR6 into AGO6 [18]. For example, RNA interference of TE mRNAs generates 21-nt and 22-nt sRNAs that are incorporated into AGO6 and direct DNA methylation [19]. Additionally, 21-nt sRNAs associated with AGO2 have been implicated in an alternative mechanism of DNA methylation targeting [20].

Although our molecular and biochemical understanding of sRNA production and RdDM is advanced, the purpose of these pathways and their function at different stages of the plant life cycle is incomplete. Despite the abundance of 24-nt sRNAs in vegetative tissues, vegetative phenotypes are not apparent for most Arabidopsis mutants in the Pol IV-RdDM pathway. However, there are clear consequences of mutations in this pathway during the reproductive stage of the life cycle. *NRPD1*, which encodes the largest subunit of Pol IV, was originally identified in genetic screens for mutants defective in transgene silencing [21]. In Arabidopsis, *NRPD1* and other RdDM components promote seed inviability in interploidy crosses [3,22–24]. Reproductive phenotypes are even more readily apparent in other species. RdDM mutants in the related species *Brassica rapa* are sterile and have severe seed defects caused by a maternal sporophytic effect [25]. Consistent with these findings, *Capsella rubella* and *Capsella grandiflora* also exhibit maternal effects in RdDM mutants [26,27]. In tomato, disruption of *NRPD1* results in abnormal embryo development and seeds that lack dessication tolerance [28]. Additionally, *Capsella rubella* exhibits post-meiotic arrest of pollen at the microspore stage and compromised TE silencing in *nrpd1* mutants [27]. Interestingly, mutations in *NRPE1* and *RDR2* do not result in pollen defects in *C. rubella* [26]. It is possible that Pol IV may still be recruited in *nrpe1* mutants and thus able to maintain some production of sRNAs, ensuring normal pollen development. In the *rdr2* mutant, other RDR factors may compensate, allowing pollen to mature normally. Here, we discuss the function of sRNAs during reproduction with a primary emphasis on 24-nt sRNAs generated by Pol IV.

## sRNA populations specific to reproductive tissues

In both vegetative and reproductive tissues, most sRNAs arise from TEs and TE fragments. Tissue and cell-type specific profiling in reproductive tissues has highlighted distinct features of reproductive sRNA populations and suggested potential functions (Table 1).

**Table 1.** Description of named small RNA classes in reproductive tissues.

Name	Tissue/ Cells	From	Number of loci	Size (nt)	Hypothesized Function
easiRNAs	Pollen	TEs	1733 in Arabidopsis [29]	21–22	**Genome Defense**: easiRNAs are loaded into AGO1, triggering cleavage of transposon transcripts and establishing PTGS [29]. **Hybridization Barriers**: Proposed to act as a quantitative sensor for paternal genome dosage [3,24].
HyperTE sRNAs	Tapetum	TEs	797 in Arabidopsis [30]	24	**Genome Defense**: sRNAs are transcribed from HyperTE loci in tapetal nurse cells and translocated into meiocytes to direct methylation patterns in sperm. These sRNAs silence transposons, thus maintaining genome integrity in the male germline [30]. **Regulation of Genes**: Methylation in *trans* of protein-coding genes in meiocytes might alter gene expression.
sirenRNAs	Ovule	Near genes	133 (*A. thaliana*) [31] 191 (*B. rapa*) [32] 801 (*O. sativa*) [33]	24	**Regulation of Developmental Genes**: A subset of siren RNAs target AGL transcription factors in endosperm. This family of TFs is essential for proper endosperm cellularization and seed development [34]. **Hybridization Barriers**: sirenRNAs might regulate response to genome dosage. Increasing ploidy of maternal parents in paternal excess crosses can alleviate dosage effects attributed to reduced dosage of maternal sirenRNAs [34]
phasiRNAs	Anthers (21 nt) Tapetum & meiocytes (24 nt)	Protein-coding regions, lncRNAs	463 (21-nt, maize) 176 (24-nt, maize) [35]	21 or 24	**Regulation of Gene Expression**: phasiRNAs may direct mRNA cleavage. *PHAS* loci are required for male fertility.

The male germline develops when meiocytes form inside anthers. Meiocytes (also known as microspore mother cells) are surrounded by a sporophytic cell layer called the tapetum, which nourishes the meiocytes and subsequent mature microspores and contributes materials to the outer layer of the mature pollen grain (Figure 1(A)). Profiling of sRNAs in meiocytes revealed that about 90% of 24-nt sRNAs were concentrated in only about 800 genomic clusters. These clusters have been mapped to hypermethylated TEs, which have been referred to as HyperTE loci (Table 1, Figure 1(A)) [30]. Interestingly, when allowing for up to three mismatches, these sRNAs imperfectly map to genes that are hypermethylated in microspore mother cells and microspores. Genetic evidence indicates that HyperTE loci are required for methylation of these genes. sRNAs derived from HyperTE loci are also enriched in tapetal cells, and the corresponding target loci are highly methylated. In contrast to meiocytes, genes with mismatches to the sRNAs are not methylated in tapetal cells [30]. The end product of male germline development is pollen, one grain of which contains a vegetative cell and two sperm cells. Epigenetically activated small interfering RNAs (easiRNAs) are Pol IV-dependent 21–22 nt sRNAs in the vegetative nucleus that are produced from TEs cleaved by microRNAs (miRNAs) [29] (Table 1). More recently, this class of sRNAs has also been described in other tissues, suggesting that easiRNAs appear prominent in pollen because 24-nt sRNAs are reduced [4,36]. easiRNAs contain the RNA modification pseudouridine [37].

On the female side, sRNAs have been profiled in ovules of *Arabidopsis thaliana* and *Brassica rapa* (Figure 1(B)). Like tapetal cells, ovules are characterized by high amounts of 24-nt sRNA production from relatively few loci (~100–200), compared to the thousands of 24-nt sRNA loci in leaves or flower buds [31,32]. Differences in 24-nt sRNA accumulation correspond to tissue-specific differences in non-CG DNA methylation [31]. However, unlike the HyperTE loci, ovule sRNA loci, which have been termed siren loci, are enriched near genes and are more likely to represent unique sequences (Table 1, Figure 1B) [31,32]. Production of siren sRNAs is dependent on *NRPD1* (Pol IV) but not on *NRPE1* (Pol V) [31,32]. Although the presence and characteristic features of siren loci are conserved among Brassica and Arabidopsis ovules, individual siren loci are not [32].

After fertilization, the ovule integuments develop into the seed coat. Profiling of seed coat sRNAs from developing seeds indicate similar patterns to that observed in whole ovules. For Arabidopsis species and *B. rapa*, the fraction of 24-nt sRNAs is reduced in the seed coat compared to other vegetative tissues, with fewer loci producing most 24-nt sRNAs. sRNAs are depleted from TEs and enriched in unique sequences [22,38]. Thus, it is likely that the patterns of sRNAs present in ovules persist in the seed coat after fertilization (Figure 1(C)).

sRNAs have also been extensively profiled in the *Arabidopsis thaliana* and *Arabidopsis lyrata* embryo and endosperm, the two products of fertilization [22]. Embryo sRNA profiles are similar to vegetative tissues, with more abundant sRNAs in heterochromatin. This is consistent with increased CHH methylation during embryogenesis in Arabidopsis and soybean [39,40] and the ability of RdDM to be restored across multiple generations [41]. Relative to the embryo, endosperm 24-nt sRNAs are shifted away from the pericentromeric heterochromatin to genic regions [22,42]. Endosperm 24-nt sRNAs from heterochromatic regions are paternally biased, consistent with higher methylation of paternally than maternally inherited genomes in the endosperm. By contrast, endosperm genic sRNAs are maternally biased [22,42]. Some endosperm sRNA loci are imprinted, meaning they are primarily expressed from one parental allele, and this is anti-correlated with transcription from parental alleles of nearby or overlapping genes [22] (Interestingly, canonical and non-canonical RdDM pathway genes are themselves paternally expressed imprinted genes in *Arabidopsis thaliana* and *Arabidopsis lyrata* endosperm, suggesting the possible importance of the pathway in this tissue [43]) Disruption of *NRPD1* in the maternal parent impacts genic endosperm sRNAs much more than paternal inheritance of *NRPD1* mutations [42,44]. This indicates a maternal sporophytic, female gametophytic, or imprinting effect of *NRPD1* on endosperm sRNAs. It is unknown whether the maternal effect of *NRPD1* is due to movement of sRNAs from maternal tissues into the endosperm (Figure 1(C)) or is an indirect effect.

Early characterizations of sRNA populations from different Arabidopsis tissues indicated a higher abundance of Pol IV-dependent sRNAs in floral and reproductive tissues compared to vegetative tissues [45–48]. The exact function of abundant sRNAs derived from relatively few loci, such as HyperTE loci and siren loci, is unclear. One hypothesis is that these sRNAs function in safeguarding the genome from TE mobilization during reproduction. It may be more efficient to generate large quantities of sRNAs from a handful of loci that are able to act in *trans*. In comparison, sRNAs in vegetative tissues may function in a maintenance role, resulting in a more even distribution of sRNA-producing loci across the genome. Another explanation is that sRNAs may mediate parent-of-origin effects. For example, maternal sRNAs derived from siren loci might dominate early seed development in order to establish gene expression programs. Alternatively, sRNAs in reproductive tissues might not have defined functions and are rather a byproduct of altered expression of RdDM pathway genes for non-RdDM functions. Furthermore, much of our current understanding of sRNAs outside of reproduction comes from studies on bulk tissue, potentially masking cell-type specific, high sRNA abundance. fFor example, the columella cells of the root cap are known to have high levels of 24-nt sRNAs and CHH methylation [49]. Given the high expression of Pol IV in roots and the reported mobility of Pol IV-dependent sRNAs originating in roots, it is possible that specific cell types in vegetative tissues have higher sRNA abundances than previously thought [50]. Future studies employing single-cell-type sequencing and spike-in normalizations will help define sRNA composition across tissues, allowing for accurate quantification and comparison.

## Mechanisms of tissue-specific sRNA production in reproductive tissues

Given the specificity of certain classes of sRNAs for certain tissues or cells (Table 1), how are tissue-specific patterns of sRNA expression generated? Several recent studies have isolated novel components of the RdDM pathway that act upstream of Pol IV. Pol IV is recruited to the sites it transcribes by a number of different factors, including the CLSY chromatin remodellers and the zinc finger protein ZMP1. Cryo-EM in combination with AlphaFold predictions identified a conserved CYC-YPMF motif that is necessary for direct interaction of CLSYs with Pol IV [51]. CLSYs are expressed in a tissue-specific manner, which contributes to tissue-specific methylation patterning [52]. CLSY1 and CLSY2 interact with SHH1, which binds to H3K9me2 and unmethylated lysine 4 of histone 3, histone modifications that are associated with transcriptional silencing. The interaction between CLSY1/2 and SHH1 is required for Pol IV sRNA transcription at > 6700 sites. In contrast, CLSY3 and CLSY4 interact with Pol IV, in a manner genetically dependent on CG methylation but not dependent on SHH1 or H3K9me2, to regulate sRNA production at >1700 loci that are largely non-overlapping with CLSY1/2-dependent loci [52].

In addition to locus-specificity, the CLSY remodellers have distinct tissue-specific roles (Figure 1). For example, *CLSY1* is expressed in leaves and required for the production of 24-nt sRNAs there. All four *CLSY* genes are expressed in flower buds, with higher expression of *CLSY3* and *CLSY4* in ovules and tapetal cells [30,31]. Correspondingly, CLSY3 controls the production of sRNAs at HyperTE and siren loci. ChIP-seq analyses revealed that CLSY3 localization at siren loci depends on a conserved DNA sequence motif [31]. The rice homolog of CLSY3 also binds specific sequence motifs, one of which has been shown to regulate DNA methylation and sRNA expression at siren loci in female reproductive tissues [53,54]. The tissue- and locus-specificity of CLSY3 is conferred in part by REM transcription factors, which are able to bind to the same DNA sequence motif *in vitro* as CLSY3 in Arabidopsis. Mutations in specific REMs selectively affect sRNA production at HyperTEs in anthers, whereas mutations in other REMs and *GDE1* alters sRNA levels at siren loci in ovules [55,56].

## Cell autonomous vs non-autonomous effects in reproductive tissues

A limitation of studying null mutants in sRNA pathway genes is that any tissue-specific or cell non-autonomous function is masked because all tissues are null for the gene of interest. Given the proximity of sporophytic, gametophytic, and offspring tissues to each other throughout reproductive development, it is critical to assess both the contributions of sRNAs individually in each tissue and the ability of sRNAs to act non-cell autonomously. Experimental strategies to approach this include tissue-specific expression of an sRNA pathway component in an otherwise null background or tissue-specific disruption of the pathway in a wild-type background.

### Male gametogenesis and surrounding tissues

Many studies have implicated RdDM in ensuring normal male gametogenesis [27,57–59]. Although gamete companion cells, such as tapetal cells and vegetative cells, do not genetically contribute to the next generation, RdDM activity in these companion cells may act to influence molecular signatures (e.g. DNA methylation, sRNA profiles and gene expression) in the gametes or in seed tissues post-fertilization.

In Arabidopsis, CLSY3 controls the expression of 24-nt sRNAs in tapetal cells over HyperTE loci. Expression of RDR2 under a tapetum-specific promoter in an *rdr2* mutant is sufficient for sRNA accumulation in microspore mother cells [30]. Combined with this result, a strong correlation between tapetal cells and meiocyte sRNA profiles suggests that sRNAs move intercellularly from the tapetum to microspore mother cells (Figure 1(A)). These sporophytically derived sRNAs could then trigger DNA methylation in microspore mother cells at genes with similar but not identical DNA sequence, thus inducing heritable epigenetic changes in the gametophytic cells. However, the extent of these effects on gene expression is not clear for many target genes [30]. In addition to transport of tapetum-derived sRNAs, movement of sRNAs between the vegetative cell and sperm in mature pollen has also been proposed. Using fluorescent reporters, Martinez et al. showed that siRNAs produced from a truncated GFP constructed under a vegetative cell promoter can silence a GFP reporter expressed in sperm [60]. However, the molecular mechanism underlying this gene silencing is unclear. In addition to studies in Arabidopsis, a recent report in *Capsella rubella* identified a population of Pol IV-dependent mobile sRNAs (PMsiRNAs) [50]. Grafting an *nrpd1* shoot onto wild-type roots was sufficient to restore pollen viability, suggesting maternal sporophytic sRNAs from the roots are able to move to male reproductive tissues to trigger formation of secondary sRNAs and post-transcriptional gene silencing [50].

### Female gametogenesis and surrounding tissues

Maternal sporophytic tissues surround the female gametophyte throughout development, making isolation of cell autonomous and cell non-autonomous effects technically challenging. During gametogenesis, the megaspore mother cell (MMC) undergoes meiosis to form four haploid spores, of which three will degenerate. The remaining haploid megaspore undergoes three mitotic divisions to form the female gametophyte (Figure 1(B)). The MMC and resulting haploid spores are encased by maternal tissues throughout these cellular divisions.

From genetic studies, RdDM factors have been shown to impact female gametogenesis. Non-canonical RdDM factors are important for specifying female germ cell identity. Proper MMC differentiation requires *SPL/NZZ*, which is upregulated in *ago9*, *rdr6*, and *drm1 drm2* mutants [61]. Furthermore, *SPL/NZZ* is required in the L2 layer of the ovule primordium but is primarily expressed in the L1 layer, indicating a non-cell autonomous dependency on RdDM [61]. Sporophytic tissues surrounding the female gametophyte are also sites of RdDM activity. Abundant levels of 24-nt sRNAs originating from siren loci are detected in the maternal seed coat [32,62]. These sRNAs can induce DNA methylation in *trans* at loci with 1 to 2 mismatches and thereby regulate gene expression [38]. However, it is unclear if these effects are primarily cell autonomous or non-autonomous. Live cell imaging in Arabidopsis egg cells revealed a requirement for DRM2 and Pol V but not Pol IV to maintain CHH methylation, suggesting that Pol IV activity within the egg cell is dispensable and that extrinsic sRNAs or one of the non-canonical RdDM pathways might act to induce methylation [63]. Movement of labelled sRNAs from the central cell to the egg cell has been observed following microinjection [64]. Consistent with this observation, silencing of a ubiquitous GFP reporter in egg cells is detected when sRNAs generating hairpins are expressed under a central cell promoter [65]. Furthermore, the sRNA profiles between egg cells and ovaries in rice are highly similar. Movement of sRNAs from the maternal sporophyte to gametophyte in rice could explain the observed similarity [66]. However, the capability for non-cell autonomous silencing in the female gametophyte seems to occur primarily prior to fertilization and is reduced post-fertilization [64,65]. Additional future experiments to directly test sRNA movement between female reproductive tissues will be required to fully explain these observations.

## Genome dosage and RdDM pathway genes

Interploidy crosses have proven to be a valuable phenotypic method for assaying the contributions of Pol IV and other RdDM factors to seed development. Crosses between plants of the same species but different ploidies often result in seed abortion due to defects in endosperm development resulting from alteration to the 2 maternal: 1 paternal genomic ratio.

*NRPD1* is required for the endosperm response to genome dosage. Paternal excess crosses (i.e. extra genomes are inherited through the sperm) between diploid and tetraploid Arabidopsis plants typically result in ~80% seed abortion, although the severity of the phenotype depends on the accession. In this genetic cross , the F1 embryos are triploid and endosperms are tetraploid, with two maternal and two paternal genomes. Under these conditions, the endosperm, which in wild-type plants develops as a coenocytic tissue and then cellularizes around the heart stage of embryogenesis, fails to cellularize and the embryo arrests. Paternal inheritance of RdDM pathway mutations, including mutations in *NRPD1*, *NRPE1*, *RDR2*, and *DRM2*, significantly represses paternal excess seed abortion and promotes viability of triploid progeny [3,22,23]. Thus, loss of paternal *NRPD1* and other RdDM pathway genes partially masks the presence of the additional paternal genome in the endosperm. Except for *nrpd1*, the ability of RdDM pathway mutants to suppress triploid seed abortion requires at least one generation of homozygosity [3,24].

In Arabidopsis, the wild-type endosperm of paternal excess crosses has reduced 24-nt sRNAs and lower levels of DNA methylation, suggesting that RdDM activity is compromised [3,23]. Why, then, does inheritance of a mutation in RdDM pathway genes suppress paternal excess seed abortion? Genetic evidence indicates that suppression of seed abortion is a paternal effect. Thus, the relevant tissue could be either paternal sporophytic and/or male gametophytic tissues or cells. In a recent study, Pachamuthu et al. expressed the gene *RTL1* under pollen-specific promoters and assayed sRNA biogenesis, DNA methylation, and interploidy seed viability. RTL1 cleaves long dsRNAs and non-specifically reduces the abundance of endogenous siRNAs processed by DCL2, DCL3, and DCL4, including those made from Pol IV precursors [[67]]. Expression of *RTL1* under a promoter active in the late microspore and the vegetative cell (pLAT52) and another promoter active in the microspore and sperm (pMGH3) causes reduced 21–22 and 24-nt sRNAs and DNA methylation from several hundred non-overlapping transposable element loci, as assayed in whole pollen. In *jas* mutants, which produce a fraction of diploid pollen and thus triploid seeds upon self-fertilization, the pLAT52 line reduced triploid seed abortion to a similar extent as mutations in *NRPD1*, from 30% to 40% seed abortion to ~20%, whereas the pMGH3 line did not reduce seed abortion [68]. These results suggest that dsRNAs present in late microspores or vegetative cells, which are presumed to be processed into sRNAs, promote interploidy seed abortion. One possibility is that the vegetative cell serves as a factory for the production of sRNAs, or their precursors, that move to and function in the sperm (Figure 1A). Another is that vegetative cell sRNAs are delivered to the endosperm precursor when the pollen tube bursts.

In contrast to paternally inherited mutations, most mutations in RdDM genes inherited through diploid mothers do not repress seed abortion in paternal excess crosses [23,44] and instead seem to further sensitize the endosperm to excess paternal dosage. Consistent with this notion, loss of maternal Pol IV function in *Capsella rubella* in the context of balanced diploid crosses results in loss of chromatin condensation and non-CG methylation in the endosperm, as well as delayed endosperm cellularization and increased seed abortion, mimicking phenotypes present in wild-type paternal excess crosses [34]. Thus, *NRPD1* has distinct maternal and paternal effects on endosperm and seed development, some of which appear to be antagonistic.

Several hypotheses have been proposed to explain the difference in phenotype severity between Arabidopsis and other plant species (Arabidopsis seed phenotypes are observed in the context of interploidy crosses, whereas in other species seed phenotypes are observed in balanced diploid crosses). RdDM may have stronger effects in species with higher TE content. Increased RdDM activity to silence TEs increases the likelihood of affecting expression of protein-coding genes, possibly giving rise to phenotypes observed in rice and tomato, which each have ~40% or more TE content in their genomes [69,70]. In contrast, TEs comprise only ~20% of the Arabidopsis genome. More recent studies have implicated parental conflict in driving the difference in RdDM phenotypes among species. RdDM acts to sense genome dosage, which is linked to the degree of conflict between maternal and paternal genomes. Under the WISO (weak inbreeder, strong outcrosser) hypothesis, the degree of parental conflict in primarily self-fertilizing species like Arabidopsis is proposed to be reduced, leading to a reduced role for RdDM in sensing genome dosage. This is consistent with mild phenotypes in Arabidopsis RdDM mutants [71]. In contrast, outbreeding species have stronger conflict and thus stronger phenotypes, such as those observed in *B. rapa* [25]. A recent comparative study between Brassicaceae species found that *rdr2* and *nrpe1* mutants have more severe phenotypes in the obligate outcrosser *C. grandiflora* compared to its recently diverged inbreeding sister species *C. rubella*, thus linking mating system and reproductive success of RdDM mutants [26]. Although the WISO hypothesis offers an explanation for phenotypic differences between RdDM mutants of different species, it is unclear why self-fertilizing species maintain the RdDM machinery, where parental conflict should be absent. However, selfing is relatively recently evolved in *Arabidopsis thaliana* and *Capsella rubella*, the self-fertilizing species in which we know most about RdDM, and *Arabidopsis thaliana* has measurable rates of outcrossing [72–76]. There might not have been sufficient evolutionary time for negative selection against regulation via RdDM, or the ability to outcross, even at a low level, promotes the retention of RdDM. Although the primary role of RdDM might be to mediate parental conflict in outcrossers, a secondary role in TE silencing might also explain the continued presence of RdDM in self-fertilizing species. Additionally, there might be key genes (e.g. the DNA demethylase gene *ROS1*) that are regulated by RdDM [77,78] that are not under conflict, thus necessitating RdDM components in self-fertilizing plants, albeit in a reduced role. Future characterization of RdDM in more ancient self-fertilizing species will help clarify the extent to which mating systems determine requirements for RdDM.

## Functions for sRNA pathway genes beyond DNA methylation

It is often presumed that the reproductive phenotypes present in *nrpd1* or other RdDM pathway mutants are due to loss of sRNAs and the ability to methylate DNA, along with consequent increases in TE expression. However, RdDM factors can have roles independent of RdDM, an important consideration when evaluating the molecular cause of mutant phenotypes. Additionally, reduced expression of the DNA demethylase gene *ROS1* in RdDM mutants in Arabidopsis can cause counter-intuitive silencing phenotypes because RdDM mutants are essentially hypomorphic for *ROS1* [77–79].

Non-RdDM functions for RdDM pathway genes have primarily been assessed in non-reproductive tissues. Pol IV-produced 21-nt and 22-nt sRNAs have been associated with AGO1 loading to direct degradation of mRNAs and thus regulate mRNA abundance [4]. The lncRNAs produced by Pol V serve as scaffolds for recruitment of AGO-siRNA complexes and ultimately DNA methyltransferases. However, Pol V transcription appears to occur broadly throughout the genome, far beyond where RdDM occurs [80]. Thus, the act of transcribing lncRNAs, or the lncRNAs themselves, could have additional functions and consequences. Recently, it was shown that lncRNAs transcribed by Pol V upstream of defence response genes are positively correlated with expression of those genes. Reduction of these Pol V-dependent transcripts with an artificial miRNA led to reduced expression of the downstream defence genes, while DNA methylation at the locus remained intact [81]. Additionally, early work showed that silencing and compaction of 5S rDNA loci on chromosome 4, in contrast to those silenced on chromosome 5, is dependent on Pol V but not on other upstream components of the RdDM pathway or the downstream DNA methyltransferase DRM2, suggesting a Pol V function independent of DNA methylation [82]. Additionally, sRNAs produced by canonical RdDM pathway components can be involved in processes other than DNA methylation. For example, a class of 21-nt sRNAs dependent on Pol IV is produced in response to DNA damage and may be involved in DNA repair [83].

Many RdDM components are multifunctional and likely interact with and regulate the chromatin landscape beyond directing DNA methylation. Reproductive tissues and cells undergo extensive chromatin reprogramming, possibly linking non-methylation functions of RdDM pathway genes (e.g. chromatin remodelling, PTGS, histone crosstalk, scaffolding and intercellular signalling) to reproductive phenotypes. These non-methylation roles may explain why phenotypes in RdDM mutants (or lack thereof in Arabidopsis) do not always correlate with methylation changes.

## Other sRNA types are abundant in male reproductive tissues

Although we have focused on 21–24 nt sRNAs produced by Pol IV, other classes of sRNAs are abundant in male reproductive tissues. Phased, secondary siRNAs, or phasiRNAs, are prominent in monocots and eudicots, though absent from legumes and Brassicaceae like Arabidopsis (Table 1) [84–86]. phasiRNAs have been likened to piRNAs in animals [87]. They are produced when a Pol II transcript from a *PHAS* locus is first cleaved by an AGO loaded with a 22-nt miRNA. The cleaved transcript is then turned into dsRNA by RDR6. DCL4 or DCL5 (a Dicer specific to monocots) then processes the dsRNA into consecutive (i.e. phased) 21 or 24-nt RNAs, respectively [88]. phasiRNAs accumulate in anthers, with 21-nt RNAs enriched pre-meiosis in epidermal cells and 24-nt phasiRNAs enriched from the start of meiosis onwards in the tapetum and meiocytes [35]. The site of the production of 24-nt phasiRNAs appears to be the tapetum, leading to the proposal that 24-nt phasiRNAs might be subsequently transported to the meiocytes via an unknown mechanism, perhaps via the plasmodesmata that connect tapetal cells and meiocytes [89,90]. In this respect, phasiRNAs may share similarities with sRNAs from HyperTE loci in Arabidopsis (Figure 1(A); Table 1). Almost all anther phasiRNAs are triggered by just two miRNAs [88,91,92]. In rice, MEL1, which corresponds to AGO5, accumulates in the cytoplasm of meiocytes prior to meiosis and binds 21-nt phasiRNAs [93]. Mutation of *MEL1* results in meiotic arrest and sterility [94]. In maize, *dcl5* mutants lack most 24-nt phasiRNAs and exhibit conditional male sterility when temperatures are 28/22°C or above during the day/night [89]. Sterility appears to be caused not by a meiotic defect but by lack of programmed cell death in the tapetum. The primary molecular function of phasiRNAs remains largely unclear.

phasiRNA targets do not appear to intersect with the targets of Pol IV-dependent 24-nt sRNAs, and whether or how the activity of one pathway might influence the other is not well understood. phasiRNA precursor transcripts primarily arise from several hundred to a few thousand intergenic non-repetitive genomic regions, unlike Pol IV and Pol V transcripts [35,91]. Another contrast is that phasiRNAs and their precursors are associated with membrane-bound polysomes, where miRNA-mediated target cleavage occurs [92,95,96]. Nevertheless, some evidence suggests that 24-nt phasiRNAs impact DNA methylation, directly or indirectly; maize *dcl5* mutants have reduced levels of CHH DNA methylation at loci that produce 24-nt phasiRNA precursor transcripts but not at loci that produce 21-nt phasiRNA precursors [97]. Interestingly, the CHH methylation at these loci in wild-type anthers increases coincident with increased production of 24-nt phasiRNAs [97].

## Conclusions

Recent findings have led to exciting new insights about the type and importance of sRNA pathways active during reproduction. Reproductive phenotypes in Arabidopsis associated with Pol IV-dependent sRNAs and RdDM are subtle, but altered genome dosage studies in Arabidopsis and other plant species offer powerful models to connect molecular profiles to morphological and developmental phenotypes. The discovery that CLSY chromatin remodellers in concert with sequence-specific transcription factors confer differential targeting of Pol IV and the production of cell-type specific sRNA populations will prompt further investigation into cell-type specific sRNA function. Recent evidence suggests that sRNAs can function non-cell autonomously in some reproductive tissues by moving into neighbouring cells or via long-distance transport (Figure 1). This evidence is stronger for male reproductive tissues than for female reproductive tissues. Tapetal cells share plasmodesmata with microspore mother cells, and the vegetative nucleus and sperm cells are closely associated, with movement of RNAs demonstrated between the vegetative nucleus and sperm. Plasmodesmata exist between cells of the female gametophyte, which could allow exchange of RNA molecules before but not after fertilization. Symplastic connections do not appear to exist between ovule tissues and the female gametophyte or among the seed coat, embryo, or endosperm after fertilization. Looking ahead, designing experiments to address whether and how sRNAs or their dsRNA precursors are transported after fertilization and the phenotypic consequences of such movement remains challenging: the cells and tissues of interest are closely associated, tracking endogenous sRNAs outside of the context of grafting between plants of different genotypes is almost impossible, and highly specific promoters are needed to drive sRNA production in one cell or cell type to observe its consequence in another. Despite these challenges, determining whether and how sRNAs are moving between reproductive tissues or vegetative tissues is a key goal for future research. Additionally, discerning whether sRNAs themselves are the effectors of phenotypes caused by Pol IV and RdDM pathway genes is essential.

## Data Availability

Data sharing is not applicable to this article as no new data were created or analysed in this study.
